# Blunted circadian variation of blood pressure in individuals with neurofibromatosis type 1

**DOI:** 10.1186/s13023-023-02766-7

**Published:** 2023-06-23

**Authors:** Ana M. Cieza Rivera, Tania Fernández-Villa, Vicente Martín, Isis Atallah

**Affiliations:** 1 Faculty of Health Sciences, Department of Biomedical Sciences, Area of Preventive Medicine and Public Health, Universidad de León, León, Spain; 2 Research Group in interactions gene- environmental and health (GIIGAS)/Institute of Biomedicine, University of León, León, Spain; 3 Epidemiology and Public Health Networking Biomedical Research Centre (CIBERESP), Madrid, Spain; 4 Division of Genetic Medicine, Lausanne University Hospital and University of Lausanne, Lausanne, Switzerland

**Keywords:** Neurofibromatosis type 1, Ambulatory monitoring blood pressure, Dipper, Non-dipper, Systolic variability, Circadian rhythm

## Abstract

**Background:**

Cardiovascular events such as myocardial infarction and stroke are life-threatening complications associated with Neurofibromatosis type 1 (NF1). As previous studies observed an association between cardiovascular events and the loss of circadian variations of blood pressure, we investigated the 24 h circadian rhythm of blood pressure (BP) in 24 NF1 patients (10 males and 14 females, with a mean age of 39.5 years ± 14 years) by using ambulatory blood pressure monitoring (ABPM).

**Results:**

Only one-third of the patient were dippers, 50% were non-dippers, and 17% were risers. Reduced variability of systolic and diastolic nocturnal blood pressure was observed in NF1 patients compared with several studies of normotensive individuals (p = 0.024). In NF1 patients, the blunted systolic nocturnal decline was significantly associated with the number of neurofibromas (p = 0.049) and the presence of a plexiform neurofibroma (p = 0.020).

**Conclusions:**

Most NF1 patients in this study showed a “non-dipper” pattern with a blunted nocturnal BP decline, which is considered an independent risk factor for cardiovascular events in normotensive and hypertensive individuals. Periodic monitoring of BP should be included in NF1 follow-up guidelines to diagnose masked hypertension or a non-dipper/riser pattern which would significantly increase the morbidity and mortality of NF1 patients to implement therapeutic strategies.

**Supplementary Information:**

The online version contains supplementary material available at 10.1186/s13023-023-02766-7.

## Introduction

Neurofibromatosis type 1 (NF1) is the most frequent genodermatosis with an incidence of 1:3,000 individuals. NF1 patients are prone to develop peripheral nerve sheath tumors such as cutaneous and plexiform neurofibromas. Other manifestations are café-au-lait spots, axillar and inguinal freckling, optic glioma, and skeletal dysplasia. Life expectancy in NF1 is limited by the increase risk of malignancy and cardiovascular events [1].

Cardiovascular disease is a frequent cause of premature death in individuals with NF1 [1] with can lead to early-onset cerebrovascular disease [2] such as hemorrhagic stroke even in young patients [3]. In the general population, potentially life-threatening cardiovascular events are clearly associated with hypertension and/or the blood pressure (BP) circadian pattern. Hypertension occurs in about 16% of NF1 patients. It can be essential or secondary to renal artery stenosis [4, 5] which is the main cause in childhood or due to a pheochromocytoma which occurs in 1% of NF1 patients, predominantly in adulthood [6]. Blood pressure follows a circadian fluctuation decreasing by approximately 10–20% during sleep (nocturnal dip) and peaking at the end of the night on arousal (morning surge) due to activation of the sympathetic nervous system [7]. When BP does not decrease by at least 10% from wakefulness to sleep, patients are considered “non-dippers”. In the general population, this is associated with an increased risk of cardiac, kidney, and vascular target organ injury [8–10]. On the other hand, “extreme dippers” are at risk of lacunar strokes and silent myocardial ischemia [11]. Patients who have excessive morning BP surges are at increased risk of cardiovascular events including stroke and myocardial infarctions [12–14]. However, patients with a nocturnal increase in BP (“reverse dippers” or “risers”) are associated with the worst prognosis for stroke and cardiac events [15, 16].

In NF1, most studies on blood pressure have been performed in children rather than in adults [17]. Data on adults are rather scarce. In this report, we aimed to study the circadian pattern of BP in a cohort of 24 NF1 adult patients by using an ambulatory blood pressure monitoring (ABPM) device and explore its association with the NF1-associated tumor burden of cutaneous and plexiform neurofibromas.

## Results

We studied 24 NF1 patients (10 males and 14 females) with a median age of 39 years (IQR 27–54 years). Table 1 summarizes the clinical data and BP parameters of the NF1 patients. Additional clinical and genetic information on NF1 patients is available in Supplementary Table 1. The tumor burden of cutaneous neurofibromas was distributed as follows 0: 16.7% (n = 4), 1–49: 66% (n = 16); ≥ 50: 16.7% (n = 4). 33% of patients harbored a plexiform neurofibroma (n = 8). On casual BP measurement, none of the patients was diagnosed with hypertension. No significant differences were noted for gender or mean body mass index and casual BP measurements.

**Table 1:** Clinical features and circadian rhythm pattern of blood pressure in Neurofibromatosis type 1 patients

	Total median (IQR)	Dipper median (IQR)	Non-dipper median (IQR)	Riser median (IQR)	p-value
	n = 24	n = 8 (33%)	n = 12 (50%)	n = 4 (17%)	
Age, y	38,5 (26.5–53.5)	44.0 (30.5–53.5)	34.5 (25.0–54.0)	33.5 (23.5–48.0)	0.709
Men, n (%)	10 (41.7)	3 (37.5)	4 (33.3)	3 (75.0)	0.328
BMI (Kg/m^2^)	23.8 (20.1–25.8)	23.7 (20.4–25.6)	23.9 (20.4–25.8)	22.5 (18.7–26.8)	0.944
Casual SBP mmHg	105.5 (95.0-110.0)	110.0 (110.0 -120.0)	100.0 (90.0 -109.0)	100.0 (95.0 -103.0)	0.085
Casual DBP mmHg	60.0 (60.0–70.0)	70.0 (60.0–75.0)	60.0 (60.0–70.0)	60.0 (55.0-64.5)	0.244
Casual PR bpm	75.0 (72.0–83.0)	80.0 (65.5–87.5)	75.0 (72.0–80.0)	76.5 (72.5–88.5)	0.839
**ABPM**					
24 h-SBP mmHg	110.5 (104.5–116.0)	113.5 (110.5–116.0)	107.5 (99.0-116.0)	109.0 (107.0-117.5)	0.443
24 h-DBP mmHg	69.5 (66.5–73.5)	71.0 (67.5–75.0)	68.5 (64.5–71.0)	71.5 (68.0–74.0)	0.563
24 h-PR bpm	73.9 (67.0–83.0)	76.0 (69.0-85.3)	69.5 (65.5–79.0)	83.5 (77.0–89.0)	0.098
Daytime SBP mmHg	114.5 (106.0-119.5)	119.0 (116.0-122.0)	110.0 (103.5-117.5)	108.0 (106.0-112.0)	0.076
Daytime DBP mmHg	72.0 (69.5–76.5)	76.0 (71.5–79.5)	71.5 (68.0–75.0)	71.0 (68.0–73.0)	0.264
Daytime PR bpm	77.0 (69.0–89.0)	78.5 (71.5–90.5)	73.5 (67.0-80.5)	88.0 (76.5–95.0)	0.197
Nocturnal SBP mmHg	105.0 (99.5-109.2)	102.5 (101.0-108.0)	102.5 (96.0-113.3)	109.0 (107.0-126.0)	0.249
Nocturnal DBP mmHg	63.5 (60.0–68.0)	63.0 (59.0–68.0)	63.5 (60.5–65.0)	71.0 (62.5–80.5)	0.362
Nocturnal PR bpm	70.2 (61.5–77.5)	69.5 (63.5–75.9)	66.0 (60.5–73.2)	77.5 (75.0–82.0)	0.095
**Systolic decline (%)**	**9.5 (5.0-11.1)**	**12.1 (10.4–13.7)**	**7.6 (5.0-9.5)**	**1.0 (0.9-12.14)**	**0.003**
**Diastolic decline (%)**	**13.5 (9.5–17.0)**	**18.6 (14.5–19.3)**	**12.8 (8.9–13.7)**	**12.0 (4.2–22.3)**	**0.030**
Pulse rate decline (%)	11.7 (6.8–14.3)	13.4 (7.7–15.8)	8.7 (5.4–12.9)	13.2 (10.7–16.5)	0.311

ABPM: ambulatory blood pressure monitoring; BMI: Body Mass Index; SBP: systolic blood pressure; DBP: diastolic blood pressure; PR: pulse rate; bpm: beats per minute

The median (IQR) 24 h BP was 110.5 (104.5–116.0)/ 69.5 (66.5–73.5) mmHg, and the median (IR) pulse rate was 73.9 (67.0–83.0) bpm. The median (IQR) nocturnal decline of systolic/diastolic and pulse rate was 9.53 (5.0-11.1)/ 13.5 (9.6–17.9) % and 11.7 (6.8–14.3) %. The 24 h ABPM identified 33% of NF1 patients as “dippers”, 50% as “non-dippers”, and 17% as “risers”. There were no differences in gender or body mass index among the three groups (“non-dippers”, “dippers” and “risers”). As expected, systolic (p < 0.003) and diastolic nocturnal decline (p < 0.03) were higher in “dippers” than in “non-dippers” or “risers”.

NF1 patients showed an increased proportion of “non-dippers” (67%) compared with previous cohorts of normotensive healthy individuals (Table 2). Thus, NF1 patients (“dippers” and “non-dippers”) seem to have a reduced nocturnal decline of systolic and diastolic BP when compared to historical cohorts of normotensive patients (Table 3).

**Table 2:** Proportion of patients with a “non-dipper” pattern in neurofibromatosis type 1 patients compared to healthy normotensive individuals reported in previous studies

	Total n	“Dippers” n (%)	“Non-dippers” n (%)	p-value
NF1 patients				
This study	24	8 (33%)	16 (67%)	
**General population**				
Ohkubo 2002 (9)	1208	807 (67%)	401 (33%)	**0.002**
Yalim 2020 (18)	196	68 (35%)	128 (65%)	1.000
Aranda 2010 (19)	10,358	5572 (54%)	4786 (46%)	**0.063**
Hermida 2011 (20)	1992	1284 (64%)	708 (36%)	**0.002**
Araujo 2018 (21)	5967	3416 (57%)	2551 (43%)	**0.022**
Yalin 2018 (22)	39	20 (51%)	19 (49%)	0.198
**All studies**	19,760	11,167 (57%)	8593 (43%)	**0.024**

NF1: neurofibromatosis type 1

**Table 3:** Mean systolic/diastolic nocturnal blood pressure in neurofibromatosis type 1 patients compared with healthy normotensive individuals reported in previous studies*

	All patients		“Dippers”	
	Mean systolic nocturnal decline mmHg	Mean diastolic nocturnal decline mmHg	Mean systolic nocturnal decline mmHg	Mean diastolic nocturnal decline mmHg
NF1 patients				
This study	8.6 ± 5.7	13.5 ± 6.2	12.5 ± 2.6	17.4 ± 3.3
**General population**				
Ohkubo 2002 [9]	13 ± 8	16 ± 8	17 ± 5	20 ± 5
Yalim 2020 [18]	Not specified	Not specified	9.8 ± 0.6	12.8 ± 0.7
Aranda 2010 [19]	Not specified	Not specified	Not specified	Not specified
Hermida 2011 [20]	Not specified	Not specified	14.4 ± 3.3	20.4 ± 5.0
Araujo 2018 [21]	11.1 ± 6.4	15.8 ± 11.1	15.5 ± 3.8	20.2 ± 8.1
Yalin 2018 [22]	Not specified	Not specified	Not specified	Not specified

*We used descriptive statistic parametric tests, mean (SD), to facilitate comparison with the previous studies in normotensive individuals. NF1: neurofibromatosis type 1

In NF1 patients, the blunted systolic nocturnal decline was significantly associated with the number of cutaneous neurofibromas (p = 0.049) and the presence of a plexiform neurofibroma (p = 0.020) (Table 4).

**Table 4:** Clinical features and 24 h blood pressure monitoring according to tumor burden (number of cutaneous neurofibromas and presence of a plexiform neurofibroma) in Neurofibromatosis type 1 patients

	Cutaneous neurofibromas				Plexiform neurofibromas		
	0	1–49	≥ 50	p-value	No	Yes	p-value
N (%)	4 (17)	16 (66)	4 (17)		16 (67)	8 (33)	
Age, y	28.0 (21.0–43.0)	38.0 (27.0–54.0)	51.0 (38.0–56.0)	0.251	39.0 (28.5–52.0)	35.5 (25.0–54.5)	0.854
Men, n (%)	3 (75.0)	6 (37.5)	1 (25.0)	0.301	7 (43.8)	3 (37.5)	0.770
BMI, Kg/m2	21.9 (19.8–24.3)	24.0 (20.4–25.8)	24.3 (20.6–27.2)	0.641	23.7 (19.5–25.6)	24.4 (21.5–26.6)	0.478
Casual SBP mmHg	105.0 (95.0-115.0)	107.0 (95.0-110.0)	100.0 (95.0-115.0)	0.907	109.0 (100.0-115.0)	100.0 (90.0-108.0)	0.276
Casual DBP mmHg	65.0 (55.0–70.0)	60.0 (60.0–70.0)	60.0 (60.0–65.0)	0.949	65.0 (60.0–70.0)	60.0 (57.5–64.5)	0.211
Casual PR	80.5 (69.5–89.0)	76.0 (69.5–81.5)	75.0 (74.0–80.0)	0.887	74.0 (66.0–80.0)	80.0 (75.0-84.5)	0.112
**ABPM**							
24 h-SBP mmHg	113.0 (107.5–116.0)	110.0 (102.5-115.5)	110.5 (102.0-119.2)	0.865	113.5 (107.0-116.0)	107.0 (99.0-110.5)	0.085
24 h-DBP mmHg	70.5 (69.0-72.5)	68.5 (65.5–73.0)	72 (65.0-77.3)	0.754	70.5 (67.5–73.5)	68.0 (65.0-72.5)	0.443
24 h-PR	66.5 (63.5–77.5)	79.0 (69.0–83.0)	73.9 (69.5–84.9)	0.521	70.5 (66.5–82.8)	81.0 (72.5–83.5)	0.358
Daytime SBP mmHg	114.0 (110.0-118.0)	114.5 (106.0-119.5)	111.5 (101.5-122.7)	0.938	116.0 (110.0-120.5)	106.0 (103.0-113.0)	0.070
Daytime DBP mmHg	74.5 (73.0-76.5)	71.5 (68.5–76.5)	72.5 (67.0-78.7)	0.553	73.0 (70.0–78.0)	71.5 (68.5–74.0)	0.407
Daytime PR	70.0 (65.0-82.5)	80.5 (70.0–89.0)	77.0 (71.0-88.5)	0.718	72.0 (69.0-87.5)	83.0 (73.0-89.5)	0.481
Nocturnal SBP mmHg	107.0 (103.0-111.5)	102.5 (97.5–109.0)	108.5 (102.0-113.3)	0.543	105.5 (100.5-110.7)	104.0 (96.5–109.0)	0.646
Nocturnal DBP mmHg	63.5 (62.0–65.0)	63.0 (59.5–68.0)	68.6 (61.5–74.1)	0.684	63.5 (61.5–68.0)	62.0 (59.0–71.0)	0.713
Nocturnal PR	61.0 (60.5–69.5)	72.5 (63.5–77.5)	70.2 (66.5–78.7)	0.473	66.0 (61.0-75.4)	74.0 (70.5–78.0)	0.177
**Systolic decline (%)**	**6.4 (3.2–8.7)**	**9.9 (8.3–13.1)**	**3.4 (0.5–7.7)**	**0.049**	**9.9 (8.1–12.5)**	**2.8 (0.9–8.7)**	**0.020**
Diastolic decline (%)	13.6 (12.3–16.1)	14.7 (11.5–19.1)	8.2 (4.0-10.8)	0.072	14.1 (12.8–18.8)	9.6 (8.1–14.9)	0.098
PR decline (%)	12.8 (6.6–15.6)	10.3 (6.7–14.2 )	10.8 (6.5–14.5)	0.967	12.4 (8.4–14.3)	7.4 (5.4–15.7)	0.327

BMI: Body Mass Index; SBP: systolic blood pressure; DBP: diastolic blood pressure; PR: pulse rate; bpm: beats per minute

## Discussion

Blood pressure follows a circadian rhythm. A normal nocturnal “dipping” profile is mainly due to a decrease in cardiac output whereas night-time systemic vascular resistance remains stable or increases. A “non-dipper” profile can be due to a diminished nocturnal decrease in cardiac output, an exaggerated increase in systemic vascular resistance or a combination of these factors [23]. However, the nocturnal BP pattern might also be influenced by other factors such as daily activity, the quality of sleep and the sleep position [23]. There are several clinical conditions known to be associated with a non-dipping pattern such as endocrine disorders (aldosteronism, hypercortisolism, pheochromocytoma, hyperthyroidism), renal dysfunction, disturbances of the autonomic nervous system (such as diabetic neuropathy or obstructive sleep apnea syndromes) or abnormal circadian melatonin secretion [23, 24]. A “non-dipper” pattern in healthy subjects is associated with elevated myocardial repolarization lability and impaired baroreflex function suggesting autonomic nervous system dysfunction [25] during the daytime which could be associated with excessive nocturnal activation of the sympathetic nervous system.

In this study, we evaluated the circadian pattern of BP in 24 NF1-individuals by using ABPM. We observed that only 1/3 of the NF1 patients exhibit the expected 10–20% of nocturnal BP decrease. Thus, NF1 patients displayed a blunted circadian variability of BP, even in individuals with a “dipper pattern”, when compared with most of the different cohorts of normotensive healthy individuals described in this article. When analyzing the data of Lama et al. [17], we found that normotensive NF1 children also displayed a reduced systolic and diastolic nocturnal drop of only 5.4% and 6.1%, respectively. This is important, as numerous studies have consistently shown an association between blunted sleep-time BP decline and the incidence of fatal and non-fatal cardiovascular events [20, 26–35]. On average a 5% decrease in the decline in nocturnal BP was associated with a 20% greater risk of cardiovascular mortality even in normotensive patients [20, 34]. Therefore, the impaired nocturnal decline of BP in NF1 patients could be responsible, at least in part, for the increased risk of cardiovascular events observed in previous reports and raises the question “which strategy should be used in NF1 normotensive patients with blunted BP circadian variations to reduce their risk of cardiovascular events?” Although none of the patients fulfilled the diagnostic criteria for hypertension on ambulatory BP measurement, one patient with a pronounced “riser pattern” (28 mmHg), at high risk of a cardiovascular event, could be diagnosed with hypertension by ABPM. Several studies suggested that ABPM is the most cost-effective strategy for confirming the diagnosis of hypertension among adults suspected of hypertension [36]. Therefore, we suggest that periodic monitoring of BP by ABPM should be included in NF1 follow-up guidelines.

Interestingly, a high proportion of non-dippers was also observed in the cohort of normotensive individuals described by Yalim et al.; half of them had even a riser pattern. This could be due to a higher mean body mass index of the cohort (around 29 kg/m^2^) and the presence of different comorbidities (diabetes, thyroid dysfunction and dyslipidemia) in 29% of individuals, which are known to reduce the nocturnal decline in BP [23, 37]. In this study, they also observed significant differences in sleep time and sleep quality among “dippers”, “non-dippers” and “risers” [18]. Reduce sleep time was associated with decreased nocturnal systolic BP variability and riser profile [18]. Several studies have previously described the impact of sleep troubles on circadian BP [38, 39]. Thus, a strong reduction of nocturnal melatonin concentrations was observed in non-dipper hypertensive patients. [24, 40].

Curiously, a previous study reported that 69% of NF1 patients were “poor sleepers” [41]. Therefore, we could hypothesize that the sleep troubles frequently observed in NF1 patients may interfere with their circadian melatonin secretion and/or nocturnal blood pressure. Neurofibromin seems to play a role in the circadian rhythm as loss of neurofibromin has been reported to disrupt circadian rhythms of locomotor activity in Drosophila [42] and to impair the 24 h calcium and the pigment-dispersing factor (PDF) cycling in mouse astrocytes [43]. However, the increased prevalence of non-dippers could also be due to autonomic dysfunction. Neurofibromatosis type 1 volunteers displayed a markedly reduced thermoregulatory capacity with a blunted reduction in diastolic and mean arterial blood pressure in response to heat stress compared to controls [44]. Of note, autonomous neuropathy is also observed in patients with diabetes or hypothyroidism leading to a higher prevalence of “non-dipper” profile in those populations. Neurofibromatosis type 1 individuals might also have abnormal vascular resistance as neurofibromin is expressed in vascular smooth muscle cells, which regulate blood pressure and hypertension [45, 46]. This alteration is produced by the involvement of vessels of any organ, but is more common in renal arteries, affecting both small and large vessels due to the remodelling of their walls [4, 5, 47].

In this study, we found that the blunted variability of BP was significantly associated with the presence of neurofibromas. Following Knudson’s two-hit theory of tumor suppressor genes, cutaneous and plexiform neurofibromas have lost the second allele of *NF1*. The absence of neurofibromin in neurofibromas could lead to abnormal transcription of different genes that could modulate BP. Riccardi et al. already described in 1981 a possible link between hypertension and large plexiform neurofibromas or increased number of cutaneous neurofibromas. They hypothesized that hypertension in NF1 patients could be due to an increase catecholamine production by the neurofibromas [48]. Interestingly, an increased in urinary fractionated metanephrines was found in 7.2% of asymptomatic NF1 patients, without abnormalities at imaging, by systematic screening for pheochromocytoma [49]. Unfortunately, authors did not describe the phenotype of the patients and they did not check for a possible association with tumor burden. However, they identified a pheochromocytoma in 7.6% of NF1 patient which is much higher than expected (1%) [6].To note, most of the NF1 patients were asymptomatic. Some other endocrinological or paracrine factors cannot be excluded. Thus, the development of large neurofibromas may cause autonomous system dysfunction by mechanical compression or by developing a neurofibromatous neuropathy, with thickening of peripheral nerves, which may lead to chronic pain, sensory loss, weakness or even palsy. Neurofibromatous neuropathy occurred in 1.3% of 600 patients with NF1. It may be caused by a diffuse neuropathic process arising from inappropriate signaling between Schwann cells, fibroblasts and perineurial cells [50]. However, despite the results found, our study is not without limitations. The small size of the cohort of NF1 patients described and the use of historical controls are the main limitations. Therefore, further studies are needed to confirm these observations in a larger number of NF1 individuals and to understand the pathophysiological mechanism and the consequences of the blunted nocturnal BP in NF1.

## Conclusion

This study observes a blunted nocturnal decline of systolic and diastolic blood pressure in NF1 patients, which might partially explain the increased risk of cardiovascular events observed in NF1 patients. Periodic monitoring of BP should be included in NF1 follow-up guidelines to diagnose masked hypertension or a non-dipper/riser pattern which would significantly increase the morbidity and mortality of NF1 patients. Therapeutic strategies in normotensive NF1 patients need to be implemented to reduce their cardiovascular risk.

## Materials and methods

### Study population

Neurofibromatosis type 1 patients were identified by using the database of the Public Health Primary Care system and the database from the Leon main Hospital (Complejo Asistencial Universitario de León). The diagnosis of NF1 in all patients was made according to the guidelines of the 1987 NIH Neurofibromatosis Conference Statement [51]. Each NF1 patient underwent a physical examination and a BP measurement (“casual BP”) in the outpatient clinic. Main clinical and genetic characteristics are available in Supplementary Table 1. None of the patients had a diagnosis of hypertension or were known to have a congenital heart defect. The tumor burden of cutaneous neurofibroma was stratified in 0, 1–49, and ≥ 50, and the presence or absence of plexiform neurofibromas.

### Ambulatory blood pressure monitoring

For ABPM we used a Microlife WatchBP O3 AMBULATORY Professional 24-hour BP Monitor (SpaceLabs, Redmond, Wash., USA) weighing 260 g (including batteries). This device employs an oscillometric method with a pressure static accuracy of ± 3 mmHg and 5% pulse accuracy. Ambulatory blood pressure monitoring was performed on regular workdays. No patients played sports or took medication during the study. The reading frequency was programmed for every 30 min from 8:00 a.m. to 10:00 p.m. (daytime) and every 60 min from 10:00 p.m. to 8:00 a.m. (night-time). Recordings were considered satisfactory for analysis if more than 75% of data were obtained.

Hypertension was diagnosed when the ABPM average systolic 24-h mean BP was at least 130/80 mmHg, the daytime systolic average is at least 135/85 mmHg and the nighttime systolic average is at least 120/70 mmHg. Circadian patterns were classified by nocturnal systolic BP fall as “extreme dipper” (≥ 20%), “dipper” (10-19.9%), “non-dipper” (0-9.9%), and “riser/reverse dipper (< 0%). We then divided the patients into three subgroups: “dippers”, “non-dippers”, and “risers”. The systolic nocturnal decline of BP was calculated as follows: (mean daily systolic blood pressure – mean nocturnal systolic blood pressure)*100 / mean daily systolic blood pressure. The diastolic and pulse rate nocturnal decline was calculated using the same formula.

### Statistical analysis

Data analysis was performed using SPSS for Windows v.25.0 software (SPSS Inc. Chicago, IL USA). Due to the limited number of patients, non-parametric tests were used. The data were expressed as median and IQR. For continuous variables, we applied Wilcoxon rank-sum tests and Kruskal-Wallis tests depending on the number of groups to be compared (2 or more than 2). For count variables, we used Fisher’s exact test (graphpad.com). The chi-square test with Yates’ correction was used for big samples. P values of < 0.05 were considered as statistically significant.

## Electronic supplementary material

Below is the link to the electronic supplementary material.

Supplementary Material 1

## Data Availability

The authors confirm that the data supporting the findings of this study are available upon request.

## List of Abbreviations

ABPM: Ambulatory Blood Pressure Monitoring
BMI: Body mass index
BP: Blood pressure
Bpm: Beats per minute
DBP: Diastolic blood pressure
IQR: Interquartile range
NF1: Neurofibromatosis type 1
PR: Pulse rate
SBP: Systolic blood pressure
