# Supplementary material for: Blunted circadian variation of blood pressure in individuals with neurofibromatosis type 1
Source: Orphanet J Rare Dis. 2023 Jun 23;18:164. doi: 10.1186/s13023-023-02766-7 (PMC10290318; doi:10.1186/s13023-023-02766-7)
Supplement: Supplementary file 1 — Supplementary Material 1 [file 13023_2023_2766_MOESM1_ESM.docx]

| ID | Age | Sex | cNF | pNF / Location | CL | F | LN | OG | Osseous features | FH | Genetic variant |
| --- | --- | --- | --- | --- | --- | --- | --- | --- | --- | --- | --- |
| 1 | 60 | M | 1 | 0 | X | 0 | X | 0 | Severe scoliosis | X | NA |
| 2 | 22 | F | 1 | Back | X | X | X | 0 | 0 | 0 | c.2379delC; p.N793fsX27 |
| 3 | 21 | M | 0 | 0 | X | X | X | 0 | Scoliosis | X | c.7012_7014delCTT; p.L2337_E2339delL |
| 4 | 54 | M | 2 | Para-tracheal | X | X | 0 | 0 | 0 | 0 | c.7012_7014delCTT; p.L2337_E2339delL |
| 5 | 22 | F | 1 | 0 | X | X | 0 | 0 | 0 | 0 | NA |
| 6 | 21 | F | 0 | 0 | X | X | X | 0 | Scoliosis | 0 | NA |
| 7 | 22 | F | 1 | Sacral plexus | X | X | 0 | 0 | 0 | 0 | c.1149C>A ; p.C383* |
| 8 | 25 | M | 1 | 0 | X | X | X | 0 | Scoliosis | X | NA |
| 9 | 48 | F | 2 | 0 | X | X | X | 0 | Scoliosis | X | NA |
| 10 | 34 | M | 1 | 0 | X | X | X | 0 | Severe kypho-scoliosis | X | c.574 C>T; p.R192* |
| 11 | 57 | M | 1 | Facial | X | X | X | 0 | 0 | 0 | NA |
| 12 | 51 | M | 0 | 0 | X | X | 0 | 0 | Kypho-scoliosis | 0 | NA |
| 13 | 34 | F | 1 | 0 | X | X | X | 0 | 0 | X | c.600_6101insA; p.T2034NfsX26 |
| 14 | 42 | M | 1 | Ankle | X | X | X | X | Tibial dysplasia, severe scoliosis | 0 | c.5606T>G; p.L1869* |
| 15 | 55 | F | 1 | Sacral plexus | X | X | X | 0 | 0 | X | NA |
| 16 | 56 | F | 1 | 0 | X | X | X | 0 | 0 | X | c.574 C>T; p.R192* |
| 17 | 43 | F | 1 | 0 | X | X | X | 0 | 0 | 0 | NA |
| 18 | 53 | F | 1 | 0 | X | X | X | 0 | 0 | X | c.3827C>T; p.R1276* |
| 19 | 32 | M | 1 | 0 | X | X | X | 0 | 0 | 0 | c.7211delC; p.A2404VfsX13 |
| 20 | 45 | F | 1 | 0 | X | X | X | 0 | Severe scoliosis | X | NA |
| 21 | 35 | M | 0 | 0 | X | X | X | 0 | 0 | 0 | NA |
| 22 | 58 | F | 2 | 0 | X | X | X | 0 | 0 | X | c.6792C>G; p.Y2264* |
| 23 | 28 | F | 2 | Temporal | X | X | X | X | Sphenoid dysplasia, kypho-scoliosis | X | c.6792C>G; p.Y2264* |
| 24 | 29 | F | 1 | Breast, lumbo-sacreal | X | X | X | 0 | 0 | 0 | c.6709C>G; p.R2237* |

The tumor burden of cutaneous neurofibromas was stratified in 0 = no neurofibromas, 1= 1-49 neurofibromas and 2≥ 50 neurofibromas. CF = café- au-lait spots, F = Freckling, LN = Lisch nodules, OG = Optic glioma, FH = Family history, NA = not available
